# Rhizosphere Bacterial Networks, but Not Diversity, Are Impacted by Pea-Wheat Intercropping

**DOI:** 10.3389/fmicb.2021.674556

**Published:** 2021-05-28

**Authors:** Barbara Pivato, Amélie Semblat, Thibault Guégan, Samuel Jacquiod, Juliette Martin, Florence Deau, Nathalie Moutier, Christophe Lecomte, Judith Burstin, Philippe Lemanceau

**Affiliations:** ^1^Agroécologie, AgroSup Dijon, INRAE, Université de Bourgogne - Université de Bourgogne Franche-Comté, Dijon, France; ^2^INRAE, UE115 Domaine Expérimental d’Epoisses, Dijon, France; ^3^IGEPP, INRAE, Institut Agro Agrocampus Ouest, Université de Rennes 1, Le Rheu, France

**Keywords:** bacterial community, biodiversity, intercropping, networks, pea, rhizosphere, wheat

## Abstract

Plant-plant associations, notably cereal-legume intercropping, have been proposed in agroecology to better value resources and thus reduce the use of chemical inputs in agriculture. Wheat-pea intercropping allows to decreasing the use of nitrogen fertilization through ecological processes such as niche complementarity and facilitation. Rhizosphere microbial communities may account for these processes, since they play a major role in biogeochemical cycles and impact plant nutrition. Still, knowledge on the effect of intecropping on the rhizosphere microbiota remains scarce. Especially, it is an open question whether rhizosphere microbial communities in cereal-legume intercropping are the sum or not of the microbiota of each plant species cultivated in sole cropping. In the present study, we assessed the impact of wheat and pea in IC on the diversity and structure of their respective rhizosphere microbiota. For this purpose, several cultivars of wheat and pea were cultivated in sole and intercropping. Roots of wheat and pea were collected separately in intercropping for microbiota analyses to allow deciphering the effect of IC on the bacterial community of each plant species/cultivar tested. Our data confirmed the well-known specificity of the rhizosphere effect and further stress the differentiation of bacterial communities between pea genotypes (Hr and hr). As regards the intercropping effect, diversity and structure of the rhizosphere microbiota were comparable to sole cropping. However, a specific co-occurrence pattern in each crop rhizosphere due to intercropping was revealed through network analysis. Bacterial co-occurrence network of wheat rhizosphere in IC was dominated by OTUs belonging to Alphaproteobacteria, Bacteroidetes and Gammaproteobacteria. We also evidenced a common network found in both rhizosphere under IC, indicating the interaction between the plant species; this common network was dominated by Acidobacteria, Alphaproteobacteria, and Bacteroidetes, with three OTUs belonging to Acidobacteria, Betaproteobacteria and Chloroflexi that were identified as keystone taxa. These findings indicate more complex rhizosphere bacterial networks in intercropping. Possible implications of these conclusions are discussed in relation with the functioning of rhizosphere microbiota in intercropping accounting for its beneficial effects.

## Introduction

Rhizosphere is a dynamic zone of interactions between microorganisms and their host plants (Hiltner, 1903; Hartmann et al., 2008). These interactions can be assimilated to a feedback loop: plants release a significant part of their photosynthates in the form of rhizodeposits, which results in the recruitment of a microbial community best adapted to the rhizosphere environment; rhizosphere microorganisms interact with each other and with the host plant, and impact plant growth, nutrition and health (Philippot et al., 2013). Rhizosphere ecology has received a great deal of attention with major progress made in understanding plant-microorganism interactions (Philippot et al., 2013; Guttman et al., 2014). They have allowed to demonstrate the specificity of the so-called rhizosphere effect at the species (Lemanceau et al., 1995; Grayston et al., 1998; Berg and Smalla, 2009; Lakshmanan et al., 2014; Tkacz et al., 2020) and even at the genotype level, for maize (Peiffer et al., 2013), soybean (Zhong et al., 2019), and medic (Pivato et al., 2007). The importance of the rhizosphere microbiota in terms of abundance, diversity and beneficial effects for the host plant has led to an holistic vision of the plant and its microbiota, rather than considering plants and microbiota as standalone entities (Hacquard and Schadt, 2015; Vandenkoornhuyse et al., 2015; Theis et al., 2016). Plant growth, development, health and fitness are mediated by plant but also microbial traits, with variations in plant phenotypes directly linked to their rhizosphere microbiota (e.g., biomass: Swenson et al., 2000; flowering time: Panke-Buisse et al., 2015). Thus, the holobiont concept has been recently proposed as encompassing the plant *per se* and its associated microbiota (Vandenkoornhuyse et al., 2015). Lemanceau et al. (2017) have further proposed the concept of functional core microbiota, in which plants recruit given microbial functional genes whatever the soils in which they are cultivated. Identification of plant and microbial traits involved in positive feedback loops has become a major target for plant-breeding in order to take better advantage of beneficial effects of rhizosphere microbiota (Wei and Jousset, 2017) for decreasing the use of chemical inputs in a more sustainable agriculture (Lemanceau et al., 2015).

Agroecology aims at valuing biotic interactions in agroecosystems in order to reduce the use of chemical inputs. A specific attention is given to crop diversification to promote agriculture sustainability (Altieri, 1999; Wezel et al., 2014; Bedoussac et al., 2015; Lemanceau et al., 2015). A classic strategy for increasing plant diversity in cropping systems is the intercropping (IC) that consists in cultivation of different plant species or cultivars on the same field and at the same time (Willey, 1979). Intercropping is a longstanding and widespread practice in low-input cropping systems throughout the world (Altieri, 1999; Knörzer et al., 2009; Maitra et al., 2021). Intercrop area represents 20-25% of arable land in China, and 17% in India, and up to 83% in Northern Nigeria, and 94% in Malawi (Knörzer et al., 2009). Indeed, in Europe, intercropping systems, such as wheat-pea, encounter different obstacles that contribute to their slow adoption and dissemination (Mamine and Farès, 2020). Varietal selection is one of the main technical limit identified by authors (Mamine and Farès, 2020).

Intercropping may allow to increasing yields (Bedoussac and Justes, 2010a; Mamine and Farès, 2020), while reducing or even avoiding the use of nitrogen fertilizers when using legumes thanks to their ability to fix atmospheric nitrogen (Pelzer et al., 2012). Indeed, legumes promote the uptake of nutrients (e.g., nitrogen, phosphorus and iron, …; Hinsinger et al., 2011; Zuo and Zhang, 2009; Xue et al., 2016) and grain protein content of the associated cereals (Bedoussac et al., 2015). Additionally, IC allows to: (i) reducing the pressure of weeds, by occupying available ecological niches, and that of pests, through the physical barrier effect (Corre-Hellou et al., 2011), and to (ii) providing mechanical support to the peas by the cereals (Bedoussac et al., 2015).

Nitrogen-fixing bacteria contribute to the above-referred beneficial effects of the associated legumes. More generally, it has been proposed that rhizosphere microbiota may account for the added value of IC. Thus, attempts have been to assess the impact of IC on rhizosphere microbiota. Total microbial communities (Taschen et al., 2017; Li et al., 2018; Gao et al., 2019; Liu et al., 2021; Tang et al., 2021) and specific functional guilds (e.g., ammonia oxidizing bacteria, Song et al., 2007; diazotrophic Proteobacteria, Solanki et al., 2020) from the total root systems of plant genotypes associated in IC have been analyzed. These reports evidenced that rhizosphere microbiota from IC and sole-cropping (SC) differ significantly, these differences being more strongly expressed for bacteria than for fungi (Gong et al., 2019). Changes in bacterial communities were mostly associated with differences in the abundance of specific phyla. These phyla were either increased (e.g., Proteobacteria, Chloroflexi, Gemmatimonatedes, Acidobacteria, Nitrospirae, and Firmicutes in proso millet and mung bean; Rhizobiales, Burkholderiales, Pseudomonadales and Bacillus populations in wheat and alfalfa; Actinobacteria in wheat and pea) or decreased (Actinobacteria in proso millet and mung bean; Sphingomonadales and Xanthomonadales populations in wheat and alfalfa, α-Proteobacteria and Acidobacteria in wheat and pea) (Taschen et al., 2017; Gong et al., 2019; Li et al., 2020). However, considering separately the roots of each plant genotypes cultivated in IC, no consistent conclusion can be drawn. No difference could be detected between microbiota from IC and SC of fababean and wheat (Tang et al., 2016), or between rhizobia populations in IC and SC of maize and soybean (Herrmann et al., 2014). In contrast, abundance of ammonia oxidizing bacteria was increased in maize and fababean in IC compared to the respective SC (Song et al., 2007). Conclusion variations between reports could possibly be ascribed to differences in compatibility between plant genotypes cultivated together. Optimization of plant-plant interactions in intercropping by an appropriate choice of plant genotypes and cultivars is a major issue. It has been hypothesized that this choice is also crucial to value beneficial plant-plant-microbe interactions. This is supported by the different responses of the root bacterial community of two sugarcane varieties when intercropped with soybean (Liu et al., 2021). Thus, identifying the appropriate plant partners in IC represent a major issue, this require to test different combinations of plant genotypes/cultivars. Biotic interactions do not only occur between plant-plant and plants-microbes, but also among microorganisms. An increasing attention is given to interaction networks between rhizosphere organisms (van der Heijden and Hartmann, 2016) and the impact of IC on these networks has recently been stressed (Liu et al., 2021; Tang et al., 2021). Thus, co-occurrence networks have been proposed as an additional parameter to characterize microbial communities (Barberán et al., 2012; Berry and Widder, 2014; Morriën et al., 2017; de Vries et al., 2018; Li and Wu, 2018; Jacquiod et al., 2020). Concerning IC co-occurrence network, Tang et al. (2021) showed, through a shotgun metagenome analysis, that sugarcane and peanut IC increased the abundance of bacterial genes involved in organic matter turnover comparing to SC, without correlating these differences to changes in microbiota diversity. Liu et al. (2021) showed differences between the co-occurrence networks between two sugarcane varieties in IC. None of these studies analyzed the differences in co-occurring network between bacterial taxa in IC and SC. These complex interactions may account for the increased yield of the IC but also of the following crop, indicating a positive legacy effect of multispecies cropping systems (Wang et al., 2020).

In the present study, we evaluated the impact of pea-wheat intercropping on rhizosphere microbiota. More specifically, we assessed how this impact may differ according to the pea and wheat cultivars. Biodiversity, structure and network of co-occurrence of bacterial community from roots of plants cultivated separately and in combination were characterized by high throughput sequencing of 16S rRNA genes. Results are discussed in the impact of IC of wheat and pea on the diversity, structure and networks of bacterial communities.

## Materials and Methods

### Site Description, Experimental Design, and Sampling

The impact of a given plant species on the rhizosphere bacterial community of the other plant species, cultivated in intercropping (IC), was tested in two independent experiments. Both experiments were performed at the Experimental Unit INRAE-Epoisses, France (47°14′11.2″ N 5°05′56.1″ E), the first from October 2016 to July 2017, the second from October 2017 to July 2018 (Table 1 and Supplementary Figure 1). Both followed a spring oat crop (Table 1). Soil physico-chemical parameters are indicated in Table 1.

**TABLE 1:** Description of first and second experiments main characteristics: year of culture, previous culture, and soil physico-chemical parameters.

		Experiment 1	Experiment 2
Year of culture		2016-2017	2017-2018
Previous culture	2016-2017	—	Spring oat
	2015-2016	Spring oat	Soft winter wheat
	2014-2015	Winter barley	Sunflower
	2013-2014	Soft winter wheat	Soft winter wheat
Soil physico-chemical parameters	Fine soil (<2 mm)	997 g/kg	996 g/kg
	Gravels (0.2-1.5 cm)	1.82 g/kg	2.32 g/kg
	Pebbles (>0.5 cm)	1.59 g/kg	1.9 g/kg
	Clay (<2 μm)	463 g/kg	403 g/kg
	Fine silt (2/20 μm)	289 g/kg	334 g/kg
	Coarse silt (20/50 μm)	187 g/kg	219 g/kg
	Fine sand (50/200 μm)	31 g/kg	25 g/kg
	Coarse sand (200/2000 μm)	30 g/kg	19 g/kg
	pH	7.81	6.87
	Total carbon (C)	19 g/kg	12.6 g/kg
	Total nitrogen (N)	1.69 g/kg	1.08 g/kg
	Calcium carbonate (CaCO)	10 g/kg	< 1 g/kg
	Phosphorus (P2O5)	0.23 g/kg	0.09 g/kg
	Calcium (Ca)	28 cmol + /kg	18.3 cmol + /kg
	Magnesium (Mg)	1.13 cmol + /kg	1.53 cmol + /kg
	Sodium (Na)	0.04 cmol + /kg	0.05 cmol + /kg
	Potassium (K)	0.56 cmol + /kg	0.45 cmol + /kg
	Iron (Fe)	0.01 cmol + /kg	0.01 cmol + /kg
	Manganese (Mn)	0.01 cmol + /kg	0.04 cmol + /kg
	Aluminum (Al)	0.03 cmol + /kg	0.04 cmol + /kg

In the first experiment, emphasis was given to the impact of wheat on pea bacterial community, by testing the effect of seven winter wheat (*Triticum aestivum*) cultivars (CF11007, CF14336, Ehogold, Flamenko, Forcali, RE13003, Renan) on the bacterial community of three winter pea (*Pisum sativum*) cultivars (cv. Fresnel - hr genotype, Geronimo and Spencer - Hr genotypes).

In the second experiment, emphasis was given to the impact of pea on wheat bacterial community, by testing the effect of 11 winter pea cultivars (Aviron, China S-29, Fresnel, Furious, Geronimo, Isard, Isard H3 1.2, Isard ttl, Joker, IVD 304/10, Spencer) on the bacterial community of two winter wheat cultivars (Ehogold and Flamenko). Each pea and wheat cultivars were cultivated in SC, and each pea cultivars was cultivated in IC with one of the two wheat cultivars. Six pea cultivars (Aviron, Fresnel, Furious, Isard, Isard H3 1.2, Isard ttl) belong to the conventional winter pea genotype hr and five (China S-29, Geronimo, Joker, Hr IVD 304/10, Spencer) to the photo-responsive winter pea genotype Hr (Table 2).

**TABLE 2:** Wheat and pea cultivars used in the present study.

		Pea genotype	Experiment 1	Experiment 2
Wheat cultivars	CF11007 = Geny		X	
	CF14336		X	
	**Ehogold**		X	X
	**Flamenko**		X	X
	Forcali		X	
	RE13003		X	
	Renan		X	
Pea cultivars	Aviron	hr		X
	China S-29	Hr		X
	**Fresnel**	hr	X	X
	Furious	hr		X
	**Geronimo**	Hr	X	X
	Isard	hr		X
	Isard H3 1.2	hr		X
	Isard ttl	hr		X
	Joker	Hr		X
	HR IVD 304/10	Hr		X
	**Spencer**	Hr	X	X

*Wheat and pea cultivars cultivated both in the first than in the second experiment are highlighted in bold.*

In both experiments, each wheat and pea cultivars were cultivated in sole cropping (SC) and in IC.

The IC set up was full mixed of the two plant species on the row IC, as previously described to be the best suitable for cereals and herbaceous legumes in intercropping (Malézieux et al., 2009). Sowing rates varied according to experimental treatments as follows: (i) wheat in SC: 300 grains/m^2^, in IC: 150 grains/m^2^; (ii) hr peas in SC: 80 grains/m^2^, in IC: 60 grains/m^2^; (iii) Hr peas in SC and IC: 40 grains/m^2^. Plants did not receive any chemical inputs, or watering. The sowing rate was optimized in order to reach 50% wheat and 50% pea at harvest in IC. In all cases, sowing rate was at 50% for wheat, that of pea differed upon cultivars. It was at 75% and 100% for hr and Hr genotypes, respectively, to take in account the difference of competitiveness of the pea genotypes (Bedoussac and Justes, 2010b).

These sowing rates allowed a plant emergence, expressed as the average ratio between IC/SC, equal to 48% for the wheat and 87% for the hr genotype, and 47,5% for the wheat and 104,5% for the Hr genotypes in the first experiment, and 62% for the wheat, 89% for the pea hr genotype, and 78% for the Hr genotypes in the second experiment.

Treatments were replicated in three blocks, each encompassing 31 plots (1.5 m × 8 m) in the first experiment and 35 in the second.

Ten root systems were randomly sampled per plot to a depth of 20 cm. In intercropping cultures, only wheat and pea root systems in close contact were sampled and their roots were further carefully separated on site. Samplings were performed at an early flowering stage for peas, which was reached 15 days earlier in hr than in Hr pea genotypes. Wheat roots were sampled at both these dates corresponding to heading stage. Bare soil was collected in three uncultivated plots integrated into blocks in each experiment and at each sampling date.

Root systems and bare soils were kept cold and transferred immediately to the laboratory. Rhizosphere soils were taken from the root systems as described by Offre et al. (2007). Samples of rhizosphere and bare soils were lyophilized at −80°C and stored at −20°C.

### Molecular Characterization of Bacterial Communities

One hundred fifty-six samples of rhizosphere soil were analyzed from the first experiment (84 from the wheat rhizosphere and 72 from the pea rhizosphere) and 177 from the second (78 from the wheat rhizosphere, 99 from the pea rhizosphere), for a total of 333 rhizosphere samples. Moreover, a total of 12 bare soil samples were also analyzed as controls, for a total of 345 samples.

DNA was extracted from soil samples (1 g dry weight) according to ISO standard 11063 (Petric et al., 2011). The library for MiSeq sequencing was generated through two PCR steps according to Berry et al. (2011). The first step consisted in amplifying all the taxa present in the samples. The bacterial 16S rRNA gene V3-V4 hypervariable region was amplified using primers Pro341F (5′-CCTACGGGAGGCAGCAG-3′) and Pro805R (5′-CCTACGGGNBGCASCAG-3′) (Takahashi et al., 2014). The PCR1 mix was prepared by adding 10X Advantage 2 PCR Buffer (Ozyme, Saint-Cyr-l’École, France), 10 mM each dNTP Mix (Thermo Fisher Scientific, Waltham, MA, United States), 10 μM of Pro805R and Pro341F each (Eurogentec, Liège, Belgium), 1.5U of 50X Advantage 2 Polymerase Mix (Ozyme, Saint-Cyr-l’École, France), 10 ng of DNA to be amplified and water up to 25 μl of final volume for each tube. Thermal cycling conditions were 2 min at 94°C, followed by 30 cycles of 30 s at 94°C, 30 s at 55°C, and 1 min at 72°C, with a final extension at 72°C for 1 min. Duplicate first step PCR (PCR1) products were pooled and then used as template for the second step PCR (PCR2). PCR2 amplification added multiplexing index-sequences to the overhang adapters using a unique multiplex primer pair combination for each sample according to Illumina guidelines. The conditions for PCR2 were the same as for PCR1, except for the number of cycles (8 cycles instead of 30).

Both PCR steps were performed on the Applied Biosystem 9700 thermal cycler (Applied Biosystem, Foster City, CA, United States). The PCRs were checked by electrophoresis (1.5% agarose, TAE1X, 100V). Two technical replicates of each PCR (1 and 2) were made, the products were then pooled and purified by Agencourt AMPure XP magnetic beads (Beckman Coulter, Brea, CA, United States), according to the supplier’s recommendations. The amplified DNA was finally quantified at StepOnePlus Real Time PCR Systems (Thermo Fisher Scientific, Waltham, MA, United States). The equimolar mixture of the samples was prepared before being sent for sequencing (GenoScreen, Lille, France). Sequencing was performed using 300-bp paired-end sequencing chemistry on the Illumina MiSeq platform (Illumina, San Diego, CA, United States). Raw paired-end reads were then demultiplexed and assembled per sample, with the Illumina MiSeq Reporter software (version 3.1).

### Bioinformatic Analyses

Sequence data were analyzed using an in-house developed Python notebook piping together different bioinformatics tools (available upon request). Briefly, quality checks of the 16S rRNA sequences were conducted using the QIIME pipeline (Caporaso et al., 2010b) and short sequences were removed (<400 bp). Reference-based and *de novo* chimera detection as well as clustering in Operational taxonomic Units (OTUs) were performed using VSEARCH (Rognes et al., 2016) and RDP representative set of 16S rRNA sequences as the reference database. The identity thresholds were set at 97%. Representative sequences for each OTU were aligned using PyNAST (Caporaso et al., 2010a) and a 16S rRNA phylogenetic tree was constructed using FastTree (Price et al., 2010). Taxonomy was assigned using UCLUST (Edgar, 2010) and the SILVA database (SILVA SSU 138 update release; Quast et al., 2012).

The raw sequences for this study have been deposited in the European Nucleotide Archive (ENA) at EMBL-EBI under accession number PRJEB42023^1^.

After cleaning, a total of 5 824 759 sequences (a mean of 16 316 sequences for each sample) were kept for OTU picking. 10 549 OTUs were delineated, and 5 713 OTUs were considered for further analysis after rarefying using the “rarefy_even_depth” function in phyloseq package.

### Statistical Analyses

Statistical analyses were conducted using R statistical software version 4.0.2 (R Development Core Team, 2014). The α-diversity of the bacterial communities was assessed by quantifying the number of OTUs per sample (richness), and by calculating the Shannon (both richness and evenness) and Simpson (evenness) indexes. β-diversity was investigated through Principal Coordinate Analysis (PCoA) of unweighted UniFrac distances. Both α and β diversity were calculated using phyloseq package (McMurdie and Holmes, 2013). Effects of plant species, cultivars, and associated plant species and cultivars on bacterial communities were tested using one- and two-way Permutational MANOVA analyses (PERMANOVA; this statistical test allows a direct additive partitioning of the variation for complex models), with 999 permutations and, when necessary, applying the ‘strata’ correction in order to restrict permutations between the 2 years of culture. Significant tests were further carried on individual pair-wise comparisons between experimental treatments, as described by Anderson (2001). PERMANOVA analyses were run using the Vegan package (Oksanen et al., 2019). OTUs explaining differences between treatments were identified by differential OTU abundance analysis. This was achieved by fitting a generalized linear model with a negative binomial distribution to normalized values for each of the OTUs and testing for differential abundance using a likelihood ratio test under DESeq package (Anders and Huber, 2010), after performing the extension DESeq with phyloseq (McMurdie and Holmes, 2014).

Wilcoxon-Mann-Whitney and Kruskal-Wallis tests were applied to identify possible significant effects on α-diversity of plant species and cultivars in monocropping and in intercropping. Kruskal-Wallis tests were followed by pair-wise comparisons with Dunn test. Wilcoxon-Mann-Whitney and Kruskal-Wallis tests were run under the dplyr package (Wickham et al., 2021) and Dunn test under the dunn.test package (Dinno, 2015).

### Network Analyses

The interactions between coexisting OTUs in rhizosphere and in bare soils were further analyzed through co-occurrence network. As suggested by Berry and Widder (2014), data of the two experiments were pooled for constructing the corresponding matrices in order to increase the size of the sample dataset and thus obtain a more stable co-occurrence network and accurate correlation estimation. Before outputting the five matrices (bare soil, SC and IC wheat, SC and IC pea), only OTUs represented in at least 50% of the samples of the entire dataset were kept for the co-occurrence network computation. Thus, OTUs only occurring in one of the two experiments were eliminated during this initial trimming, in order to keep only the common to the two experiments. The five correlation matrices amongst OTUs were calculated using Poisson Log Normal models (PLN, Chiquet et al., 2020). The models were validated by using the Bayesian Information Criterion (BIC, only r2-values provided here) and the significance of partial correlations was evaluated by a resampling of each matrix (*n* = 30) to test the robustness of the networks, using the Stability Approach to Regularization Selection (StARS) method (Liu et al., 2010). StARS method was developed for high dimensional problems and is based on random subsamples (30 iterations in our study, as stated before) and the construction of an highly stable graph from subsamples, in order to evaluate the robustness of the network along the path of solutions (Liu et al., 2010). Moreover, StARS method also allowed reducing possible biais in OTUs relative abundances between the two experiments, through the use of partial correlations. Hereafter we elaborated a network approach based on edge arithmetic (Jacquiod et al., 2020) to identify OTU correlations that were specific of intercropping (Supplementary Figure 2). Briefly, in the matrix of the pea and the wheat intercropping, we systematically removed all correlation interferences that were attributed to (i) the bare soil, (ii) the pea monocropping, and (iii) the wheat monocropping (Supplementary Figure 2). This resulted in two trimmed networks of OTUs showing specific links in the pea and in the wheat intercropping rhizosphere, respectively. Then, we intersected these two networks in order to retain the unique fractions of wheat, the unique fraction of pea, and the common conserved links found in both rhizospheres only in intercropping context. Complexity of networks was investigated by means of the degree index, the node betweenness and the edge betweenness (Newman, 2003).

## Results

### Effect of Plant Species and Cultivars Grown in SC on the Rhizosphere Bacterial Communities

The effects of the plant species and cultivars on the rhizosphere bacterial communities were analyzed on either the separate or the pooled dataset of the two experiments.

Microbial α-diversity was significantly higher in wheat than in pea rhizosphere in the first experiment including seven wheat cultivars (Wilcoxon-Mann-Whitney, *p* = 7.46e-04; *p* = 3.3e-04; *p* = 1.27e-03 for Observed, Shannon and Simpson diversity indices, respectively), but not in the second only including two wheat cultivars (Supplementary Figure 3A). In the pooled dataset, microbial α-diversity was significantly higher in wheat than in pea rhizosphere (Wilcoxon-Mann-Whitney, *p* = 3.65e-06; *p* = 1.08e-05; *p* = 6.52e-05 for Observed, Shannon and Simpson diversity indices, respectively) (Supplementary Figure 3B).

β-diversity was significantly different between wheat and pea rhizosphere in both experiments (separate datasets, PERMANOVA analysis; F-model = 2.61, *p* = 0.001, based on 999 permutations; pooled datasets, PERMANOVA analysis; F-model = 3.11, *p* = 0.001, based on 999 permutations) (Supplementary Figure 3C). Differences between the species in the pooled data sets were explained by five OTUs of Proteobacteria, three Alphaproteobacteria (two Rhizobiales and one Sphingomonadales orders) and two Betaproteobacteria (Burkholderiales order) that were preferentially associated with pea (Supplementary Data Sheet 1).

Within wheat species, no significant differences between cultivars were detected in microbial α- and β-diversity in the first experiment (Supplementary Figures 4A,B). Within pea species, α-diversity was significantly higher in Hr than in hr cultivars in the second experiment (Supplementary Figures 5A,B; Wilcoxon-Mann-Whitney, *p* = 9.8e-04; *p* = 8.59e-04; *p* = 5.67e-03 for Observed, Shannon and Simpson diversity indices, respectively). β-diversity also differed significantly between Hr and hr cultivars (Supplementary Figure 5C; PERMANOVA analysis; F-model = 1.38, *p* = 0.001, based on 999 permutations). These differences were significantly most expressed when comparing Aviron and Furious (hr) with Geronimo (Hr) cultivars (Supplementary Figure 5C PERMANOVA analysis; F-model = 1.07, *p* < 0.02, based on 999 permutations).

### Compared Effects of SC and IC on the Diversity of the Rhizosphere Bacterial Communities

**FIGURE 1:**
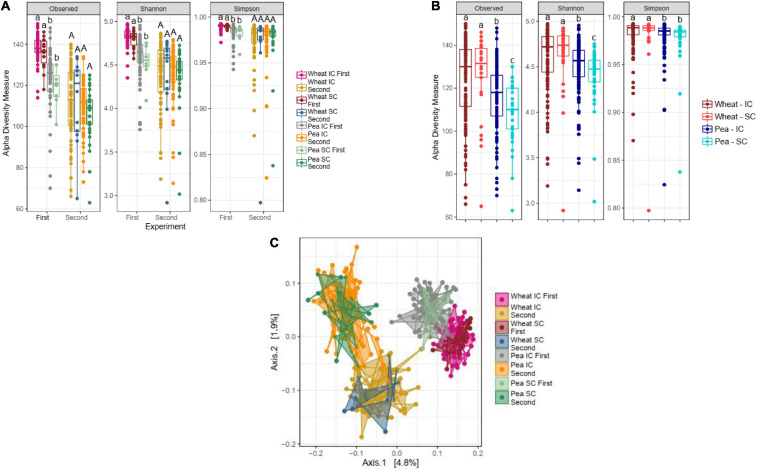
Impact of the sole- (SC) and intercropping (IC) on rhizosphere microbiota. **(A)** Box-plots illustrate α-diversity indices (Observed, Shannon and Simpson) in bacteriobiota of wheat and pea cultivated in SC- and IC, in the first and the second experiment. Median values and interquartile ranges are indicated in the plots. Different letters indicate significant differences according to Wilcoxon-Mann-Whitney test. **(B)** Box-plots illustrate α-diversity indices (Observed, Shannon and Simpson) in bacteriobiome of wheat and pea cultivated in SC and IC, in a dataset pooling results from the two experiments. Median values and interquartile ranges are indicated in the plots. Different letters indicate significant differences according to Wilcoxon-Mann-Whitney test. **(C)** PCoA (with unweighted UniFrac), representing the β-diversity results of rhizosphere microbiota profiles of wheat and pea cultivated in sole- and intercropping, in the first and the second experiment.

α-diversity of wheat bacterial communities did not differ between SC and IC with pea in any of the two experiments (Figure 1A), nor in the pooled dataset (Figure 1B). Similarly, β-diversity of wheat bacterial communities did not differ in SC and IC (Figure 1C).

α- and β-diversity of pea bacterial communities did not either differ in SC and in IC in any of the experiments (Figures 1A,C), although significant differences were detected in the pooled dataset, in which the richness of the bacterial communities associated to IC pea plants was higher than the one associated to SC pea plants (Figure 1B, Kruskal-Wallis, *p* = 2.35e-09, *p* = 5.26e-08, *p* = 3.05e-06, for Observed, Shannon and Simpson, respectively).

In order to better explore the impact of the IC on the β-diversity of wheat and pea bacterial communities, a PERMANOVA with two covariate (plant species –wheat or pea- and culture –SC or IC-) has further been performed. In all cases (separate and pooled experiments), this test confirmed that only plant species had a significant impact on β-diversity (PERMANOVA analysis for plant species factor; *p* = 0.001, based on 999 permutations).

No differences in α- and β-diversity was either detected between intercropping and monocropping when testing a data subset only including cultivars which were shared in the two experiments (i.e., wheat: Ehogold and Fresnel, and pea: Fresnel, Geronimo and Spencer).

### Effect of Mono- and Intercropping on the Co-occurrence Network of the Rhizosphere Bacterial Communities

Co-occurrence networks were produced from the pooled dataset, as a high number (>25) of samples is required to obtain a stable network with accurate correlation estimation, as recommended by Berry and Widder (2014).

The BIC R^2^ (0.98 for monocropped and intercropped wheat, 0.99 for monocropped and intercropped pea, and 0.97 for bare soil) clearly showed that the PLN models fitted the dataset, resulting in accurate correlation matrices.

After removing the edges observed in the bare soil and in the SC rhizospheres (Supplementary Figure 2), we obtained cleaned intercropping networks featuring edges only present in the rhizosphere of each IC plant rhizosphere.

The resulting cleaned intercropped wheat microbial network consisted of 573 nodes (OTUs) and 1673 edges (1462 positive and 211 negative edges; mean degree or node connectivity 5.8). The mean node and edge betweenness centrality were 851.4 and 389.6 respectively. The resulting cleaned intercropped pea microbial network consisted of 451 nodes (OTUs) and 1189 edges (1112 positive and 77 negative edges; mean degree or node connectivity 5.3). The mean node and edge betweenness centrality were 716.4 and 357.1 respectively. Pea microbial network showed a higher positive to negative edge ratio in comparison to wheat network (14.4 vs. 6.9 respectively, Supplementary Figure 6A). Mean degree, node and edge betweenness were significantly higher in wheat than pea network (Wilcoxon-Mann-Whitney, *p* < 0.05; Supplementary Figures 6B-D). 50% of the OTUs in wheat and pea networks were affiliated to Acidobacteria, Bacteroidetes and Alphaproteobacteria phyla (Supplementary Figure 6E).

We then applied a network intersection (Supplementary Figure 2) between the pea and wheat networks under IC to specifically identify: (i) the unique fraction of the pea network that was only seen in the pea under IC; (ii) the unique fraction of the wheat network that was only seen in the wheat under IC; and (iii) the common network that was shared amongst both rhizosphere under IC.

**FIGURE 2:**
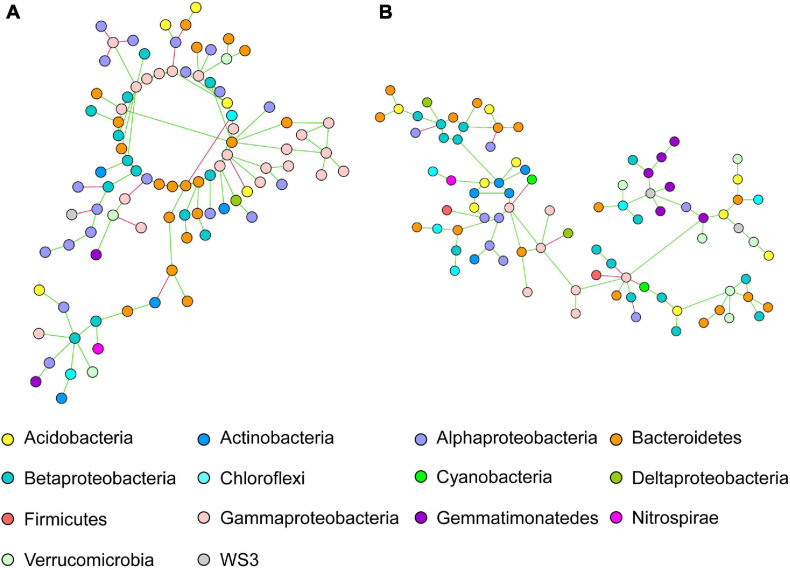
Co-occurring bacterial network of OTUs belonging to intercropped **(A)** wheat and **(B)** pea rhizosphere network. Each network node (individual circle) represents an OTU. Network edges are represented as straight lines connecting the nodes and indicate significant co-occurrences based on partial correlation obtained from a Poisson Log Normal model (*r* > | 0.06|, *p* < 0.05, *n* = 30 iterations); green for positives and red for negative co-occurrence.

The network of intercropped wheat was characterized by a dominance of Alphaproteobacteria, Bacteroidetes and Gammaproteobacteria OTUs (Figure 2A); but that intercropped pea did not show any significant taxonomic dominance (Figure 2B).

**FIGURE 3:**
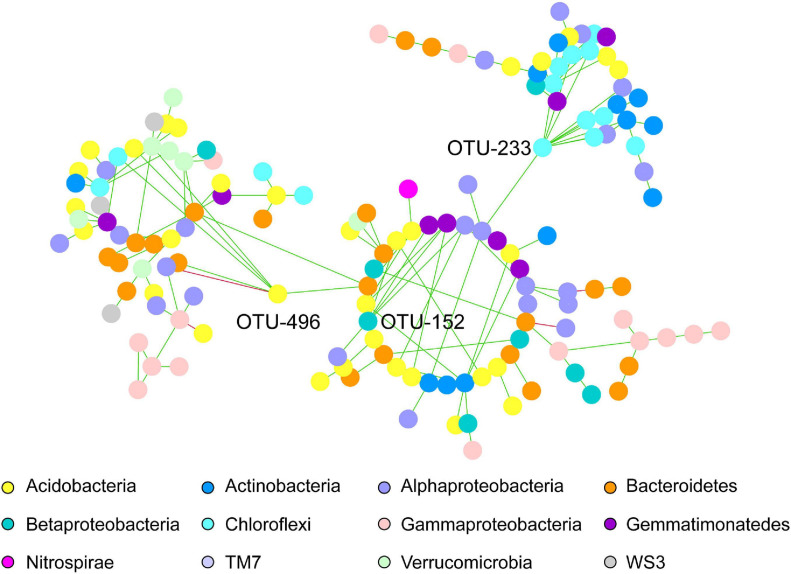
Co-occurring bacterial network of common OTUs belonging to intercropped wheat and pea rhizosphere. Each network node (individual circle) represents an OTU. Network edges are represented as straight lines connecting the nodes and indicate significant co-occurrences based on partial correlation obtained from a Poisson Log Normal model (*r* > | 0.03|, *p* < 0.05, *n* = 30 iterations); green for positives and red for negative co-occurrence. OTU-496, OTU-152 and OTU-233 have been identified as keystone OTUs.

Regarding the common network fraction shared amongst both pea and wheat rhizosphere under IC, a clear organization in modules was observed, with three keystone OTUs that had a very strong degree compared to the others. Networks belonging to intercropped wheat and pea shared three main modules including OTUs assigned to Acidobacteria, Alphaproteobacteria and Bacteroidetes phyla (Figure 3). Three keystone OTUs were further identified (Figure 3): OTU-496 belonging to Acidobacteria, OTU-152 belonging to Betaproteobacteria (order Burkholderiales, family Alcaligenaceae) and OTU-233 belonging to Chloroflexi (order Thermomicrobia).

## Discussion

An increasing attention is given to intercropping in agroecology to better value resources and to decrease the use of synthetic inputs (i.e., fertilizers, pesticides). In wheat-pea intercropping, reports indicated the promotion of nitrogen nutrition of wheat (Ghaley et al., 2005; Bedoussac and Justes, 2010a; Guiducci et al., 2018) and suggested a promotion of iron nutrition for pea (Zuo and Zhang, 2009), and of phosphorus nutrition for both plant species (Li et al., 2003, 2007; Hinsinger et al., 2011). The possible contribution of rhizosphere microbiota of plants to these beneficial effects mostly remains to be untapped. This requires to disentangle the complex interactions between the plants grown in association and their rhizosphere microbiota. A major issue is to determine whether the rhizosphere microbiota of the plant species cultivated together differ or not from that of the plant species cultivated separately, and how the IC impact would vary upon the cultivars chosen to be cultivated together. For this purpose, we have compared the rhizosphere bacterial communities of wheat and pea when cultivated in intercropping and in sole cropping, and have tested different cultivar combinations. Bacterial communities were characterized on the basis of their biodiversity, structure and of co-occurrence network of 16S rRNA genes.

In sole cropping, we confirmed the specificity of the rhizosphere effect, which was reported for long (Lemanceau et al., 1995; Grayston et al., 1998; Berg and Smalla, 2009; Lakshmanan et al., 2014; Tkacz et al., 2020). Indeed, bacterial communities from wheat and pea differed significantly (Supplementary Figure 3). Both richness and evenness of bacterial OTUs were higher in wheat than in pea rhizosphere, this greater number of bacterial OTUs is in agreement with previous reports (Turner et al., 2013; Taschen et al., 2017; Cordero et al., 2020). Differences in richness and evenness were at least partly explained by a higher representation of Proteobacteria in pea rhizosphere, especially Comamonadaceae, Sphingomonadaceae, but also, as expected, Bradyrhizobiaceae and Rhizobiaceae (Supplementary Data Sheet 1), known to be leguminous-associated bacteria (Wielbo, 2012; Chaudhari et al., 2020). Differences were less clear-cut at the cultivar level. In wheat, no difference could be detected between the rhizosphere microbiota of the tested cultivars (Supplementary Figure 4). This is in agreement with Corneo et al. (2016), Simonin et al. (2020). However, Kavamura et al. (2020) identified differences of richness between tall and semi-dwarf cultivars, which contrast with the lack of differences recorded in the present study between the four tall cultivars (RE 13 088, CF 14 336, RE 13 003 and Ehogold) and the three semi-dwarf (Flamenko, Forcali, and Renan) tested. In pea, we detected a significant higher richness of the bacterial communities in Hr than in hr genotypes (Supplementary Figure 5). This observation is consistent with the physiological differences between the genotypes known for their differential sensitivity to photoperiod, that leads to a later floral initiation (Murfet, 1973; Alcalde et al., 1999), flowering and maturity in Hr genotypes. Still, to our knowledge, this is the first report pinpointing significant discriminating effect of Hr/hr genotypes on their rhizosphere bacterial communities.

In intercropping, biodiversity and structure of rhizosphere bacterial community of plant species/cultivars did not differ significantly from sole cropping in any of the two experiments (Figure 1). This is consistent with previous reports made on wheat-fababean IC (Tang et al., 2016) and on sugarcane varieties IC (Liu et al., 2021). Differences between intercropping and sole cropping were only detected in pea, when pooling data from the two experiments. Then, bacterial community richness appeared to be higher in intercropping than in sole cropping. Our observation is consistent with previous research on intercropping involving other plant species (Li and Wu, 2018).

However, in overall, our results on biodiversity and structure are not in favor of a differential effect of IC compared to SC on wheat and pea bacterial communities. This would suggest that both plant species have similar impact on their bacteria independently of their neighboring plant. This is in agreement with Tkacz et al. (2020), who showed that bacterial community is influenced more by the root fraction than by the soil or plant species. However, the lack of differences between IC and SC could also be ascribed to the characterization methods of the bacterial community. Indeed, they provided information on the taxonomic composition and diversity, but not on the interactions between microbial groups or on their functions. Pivato et al. (2017) previously reported that despite their low impact on the total bacterial community, combination of plant species had a significant effect on the functional bacterial community mediating nitrification. Thus, additional analyses were performed to compare the co-occurrence networks in IC and SC. More specifically; we searched for the possible existence of specific co-occurrence links amongst rhizosphere OTUs that would only be recorded in IC. After filtering for all potential interference sources in our data (Supplementary Figure 2), we identified three networks whose edges were only observed in IC, only found in wheat cultivated in IC, another specific to pea cultivated in IC and finally a common network for wheat and pea cultivated in IC.

The specific wheat IC co-occurrence network was characterized by a dominance of OTUs belonging to Alphaproteobacteria, Bacteroidetes and Gammaproteobacteria (Figure 2). Alphaproteobacteria (e.g., Rhizobiales order) are known to be well represented in wheat rhizosphere (Bartoli et al., 2020), both in wild and domesticated cultivars, and thus to show a high heritability (Spor et al., 2020). Higher abundance of Bacteroidetes and Proteobacteria (e.g., Alphaproteobacteria, Gammaproteobacteria) was reported in tall than in semi-dwarf wheat cultivars (Kavamura et al., 2020). Connector bacterial OTUs belonging to Gammaproteobacteria, Alphaproteobacteria and Bacteroidetes were previously shown to be more represented in wheat rhizosphere than in bulk soil (Fan et al., 2018). The rationale for the further increase of these nodes recorded here in intercropping remained to be investigated. In the specific pea IC co-occurrence network, no dominant OTUs nodes were observed. Larger and stronger networks in bacterial communities were also described in pea when combining two cultivars (Horner et al., 2019). Lastly, wheat and pea IC specific networks had three common main modules with dominant OTUs belonging to Acidobacteria, Alphaproteobacteria and Bacteroidetes phyla. Three additional keystone OTUs were identified to be shared in networks of wheat and pea cultivated in intercropping: OTU-496, OTU-152, OTU-233, belonging to Acidobacteria (class RB41, order Ellin6075), Betaproteobacteria (order Burkholderiales, family Alcaligenaceae), Chloroflexi (order Thermomicrobia). Acidobacteria was described to be a key taxa in microbiota network associated with wheat (Kavamura et al., 2020), and among this taxa, order Ellin6075 to be part of the core microbiota of *Brassica napus* rhizosphere (Taye et al., 2020). A lot of attention has recently been dedicated to Acidobacteria in rhizosphere ecology (da Rocha et al., 2013; Kielak et al., 2016a, b; Kalam et al., 2020) with populations of Acidobacteria enriched in the rhizosphere (da Rocha et al., 2013; Kielak et al., 2016b). Genomic and metagenomic analyses allowed Kielak et al. (2016a) to predict a range of activities (e.g., the ability to attach roots thanks to exopolysaccharide production, promotion of plant iron uptake, indole-3-acetic acid production) in Acidobacteria with populations beneficial to the host-plant (Afzal et al., 2019). Acidobacteria have frequently been described as co-occurring with Proteobacteria, however it is not yet clear if this co-occurence stems from overlapping niches and/or from metabolic interactions. Alcaligenaceae have been identified as being associated with soil suppressiveness to soilborne diseases (Chapelle et al., 2016; Gómez Expósito et al., 2017). Abundance of Chloroflexi, known for their ability to oxidize nitrites, varies in wheat rhizosphere upon N addition (Ma et al., 2020). Since legumes may transfer ammonium from nodules to the surrounding soil and plants (Zhang et al., 2017), wheat intercropped with pea may benefit from an increased content in ammonium that would promote nitrifiers. Indeed, *amoA* genes appear to be more represented in maize-peanut intercropping than in maize monocropping. Among Chloroflexi, Thermomicrobia are dominant taxa in wheat rhizosphere (Latif et al., 2020) and key taxa in microbial network associated with tall wheat cultivars (Kavamura et al., 2020).

In conclusions, the present study shows that bacterial communities associated with wheat and pea differ between IC and SC, despite the lack of significant differences of their biodiversity and structure. Among the key taxa of specific of IC networks, some could be candidate promoting plant growth, nutrition and health. Our data also point out more complex networks within bacterial communities in the IC rhizosphere of wheat and pea, whereas their biodiversity and structure were not impacted. Co-occurring networks of plant microbiome were described to be more structured and complex in rhizosphere than in bare soil (van der Heijden and Hartmann, 2016). How this increased complexity may account for the beneficial effects of the intercropping on the plant growth and nutrition remains to explored. Still, recent studies clearly showed an increased function expression in belowground communities when the networks of co-occurence between populations were more complex, despite the lack of biodiversity variations (Morriën et al., 2017). The possible enhancement of functionalities in more complex microbial networks could be assumed to be related to modified activities of the populations when closely interacting. This hypothesis is currently being tested with the help of transcriptomic approaches in synthetic bacterial communities.

## Data Availability Statement

The datasets presented in this study can be found in online repositories. The names of the repository/repositories and accession number(s) can be found in the article/Supplementary Material.

## Author Contributions

BP, JB, and PL conceived the study. TG, NM, CL, and JB designed the field experiments. TG and JM performed the field experiments. BP and FD performed the sampling and were involved in the experiments in molecular biology. BP and AS performed bioinformatics and statistical analysis. BP and SJ conceived the analysis of the co-occurrence networks. SJ performed the analysis of the co-occurrence network and revised the manuscript. BP and PL wrote and revised the manuscript. All authors contributed to the article and approved the submitted version.

## Conflict of Interest

The authors declare that the research was conducted in the absence of any commercial or financial relationships that could be construed as a potential conflict of interest.

## Footnotes

1 https://www.ebi.ac.uk/ena/browser/view/PRJEB42023

## Supplementary Material

Supplementary Figure 1 Plan of the Experimental Unit INRAE-Epoisses, France (47°14’11.2″ N 5°05’56.1″ E) in panels **(A)** 2016-2017 and **(B)** 2017-2018. Blue rectangles with red outline indicate the experimental site in panels **(A)** 2016-2017 and **(B)** 2017-2018. The dark green circles with black outline indicate the sampling site for soil physical-chemical analyses.

Supplementary Figure 2 Tailored approach to identify OTU correlations that are specific of the intercropping.

Supplementary Figure 3 Impact of wheat and pea on rhizosphere bacterial community. **(A)** Box-plots illustrate α-diversity indices (Observed, Shannon, and Simpson) in bacterial communities of wheat (red) and pea (light blue) rhizosphere in the first and the second experiments. Median values and interquartile ranges are indicated in the plots. Different letters indicate significant differences according to Wilcoxon-Mann-Whitney test. **(B)** Box-plots illustrate α-diversity indices (Observed, Shannon and Simpson) in bacteriobomes of wheat (red) and pea (light blue) rhizosphere corresponding to the pooled dataset of information from the first and second experiments. Median values and interquartile ranges are indicated in the plots. Different letters indicate significant differences according to Wilcoxon-Mann-Whitney test. **(C)** PCoA (with unweighted UniFrac) showing the β-diversity results of rhizosphere bacterial community profiles of wheat and pea rhizosphere ine the first (First) and the second (Second) experiment.

Supplementary Figure 4 Impact of the wheat cultivars in sole cropping on rhizosphere bacterial community. **(A)** Box-plots illustrating α-diversity indices (Observed, Shannon, and Simpson) in bacterial community of wheat cultivars in the first experiment. Median values and interquartile ranges are indicated in the plots. **(B)** PCoA (with unweighted UniFrac) showing the β-diversity results of rhizosphere microbiota profiles of wheat cultivars corresponding to the first experiment.

Supplementary Figure 5 Impact of the pea cultivars in sole cropping on rhizosphere bacterial community. **(A)** Box-plots illustrate α-diversity indices (Observed, Shannon and Simpson) in bacterial community of pea cultivars in the second experiment. Median values and interquartile ranges are indicated in the plots. **(B)** Box-plots illustrating α-diversity indices (Observed, Shannon and Simpson) in bacterial community of pea hr and Hr genotypes corresponding to the pooled dataset. Median values and interquartile ranges are indicated in the plots. Different letters indicate significant differences according to Wilcoxon-Mann-Whitney test. **(C)** PCoA (with unweighted UniFrac) showing the β-diversity results of rhizosphere microbiota profiles of pea cultivars corresponding to the first experiment.

Supplementary Figure 6 Comparison of the architecture characteristics of co-occurring network between intercropped wheat and pea networks: **(A)** positive to negative edge ratio, **(B)** mean degree, **(C)** node betweenness, and **(D)** edge betweenness. Significant differences were indicated by ^∗∗∗^, according to Wilcoxon-Mann-Whitney test (*p* < 0.05). **(E)** OTUs affiliations in intercropped wheat and pea networks.

## References

[B1] Afzal I. Zabta K. S. Shomaila S. Shaheen S. (2019). Plant beneficial endophytic bacteria: mechanisms, diversity, host range and genetic determinants. *Microbiol. Res.* 221 36–49. 10.1016/j.micres.2019.02.001 30825940

[B2] Alcalde J. A. Wheeler T. R. Summerfield R. J. (1999). Flowering genes and the photothermal flowering responses of pea (Pisum sativum L.)-a reanalysis. *Aust. J. Plant Physiol.* 26 379–386.

[B3] Altieri M. A. (1999). Applying agroecology to enhance the productivity of peasant farming systems in Latin America. *Environ. Dev. Sustain.* 1 197–217. 10.1023/A:1010078923050

[B4] Anders S. Huber W. (2010). Differential expression analysis for sequence count data. *Genome Biol.* 11:R106. 10.1186/gb-2010-11-10-r106 20979621 PMC3218662

[B5] Anderson M. J. (2001). A new method for non-parametric multivariate analysis of variance: non-parametric manova for ecology. *Austral Ecol.* 26 32–46. 10.1111/j.1442-9993.2001.01070.pp.x

[B6] Barberán A. Bates S. T. Casamayor E. O. Fierer N. (2012). Using network analysis to explore co-occurrence patterns in soil microbial communities’. *ISME J.* 6 343–351. 10.1038/ismej.2011.119 21900968 PMC3260507

[B7] Bartoli C. Boivin S. Marchetti M. Gris C. Gasciolli V. Gaston M. (2020). Rhizobium leguminosarum symbiovar viciae strains are natural wheat endophytes and can stimulate root development and colonization by arbuscular mycorrhizal fungi’. *bioRxiv* [Preprint] 10.1101/2020.08.07.241844 35920038

[B8] Bedoussac L. Journet E. P. Hauggaard-Nielsen H. Naudin C. Corre-Hellou G. Jensen E. S. (2015). Ecological principles underlying the increase of productivity achieved by cereal-grain legume intercrops in organic farming. A review. *Agron. Sustain. Dev.* 35 911–935. 10.1007/s13593-014-0277-7

[B9] Bedoussac L. Justes E. (2010a). Dynamic analysis of competition and complementarity for light and N use to understand the yield and the protein content of a durum wheat-winter pea intercrop. *Plant Soil* 330 37–54. 10.1007/s11104-010-0303-8

[B10] Bedoussac L. Justes E. (2010b). The efficiency of a durum wheat-winter pea intercrop to improve yield and wheat grain protein concentration depends on N availability during early growth’. *Plant Soil* 330 19–35. 10.1007/s11104-009-0082-2

[B11] Berg G. Smalla K. (2009). Plant species and soil type cooperatively shape the structure and function of microbial communities in the rhizosphere: plant species, soil type and rhizosphere communities. *FEMS Microbiol. Ecol.* 68 1–13. 10.1111/j.1574-6941.2009.00654.x 19243436

[B12] Berry D. Mahfoudh K. B. Wagner M. Loy A. (2011). Barcoded primers used in multiplex amplicon pyrosequencing bias amplification. *Appl. Environ. Microbiol.* 77 7846–7849. 10.1128/AEM.05220-11 21890669 PMC3209180

[B13] Berry D. Widder S. (2014). Deciphering microbial interactions and detecting keystone species with co-occurrence networks’. *Front. Microbiol.* 5:219. 10.3389/fmicb.2014.00219 24904535 PMC4033041

[B14] Caporaso J. G. Bittinger K. Bushman F. D. DeSantis T. Z. Andersen G. L. Knight R. (2010a). PyNAST: a flexible tool for aligning sequences to a template alignment. *Bioinformatics* 26 266–267. 10.1093/bioinformatics/btp636 19914921 PMC2804299

[B15] Caporaso J. G. Kuczynski J. Stombaugh J. Bittinger K. Bushman F. D. Costello E. K. (2010b). QIIME allows analysis of high-throughput community sequencing data. *Nat. Methods* 7 335–336. 10.1038/nmeth.f.303 20383131 PMC3156573

[B16] Chapelle E. Mendes R. Bakker P. H. A. M. Raaijmakers J. M. (2016). Fungal invasion of the rhizosphere microbiome. *ISME J.* 10 265–268. 10.1038/ismej.2015.82 26023875 PMC4681858

[B17] Chaudhari D. Rangappa K. Das A. Layek J. Basavaraj S. Kandpal B. K. (2020). Pea (Pisum Sativum l.) plant shapes its rhizosphere microbiome for nutrient uptake and stress amelioration in acidic soils of the north-east region of India’. *Front. Microbiol.* 11:968. 10.3389/fmicb.2020.00968 32582047 PMC7283456

[B18] Chiquet J. Mariadassou M. Robin S. (2020). The poisson-lognormal model as a versatile framework for the joint analysis of species abundances’. *bioRxiv* [Preprint] 10.1101/2020.10.07.329383

[B19] Cordero J. de Freitas J. R. Germida J. J. (2020). Bacterial microbiome associated with the rhizosphere and root interior of crops in Saskatchewan, Canada. *Can. J. Microbiol.* 66 71–85. 10.1139/cjm-2019-0330 31658427

[B20] Corneo P. E. Suenaga H. Kertesz M. A. Dijkstra F. A. (2016). Effect of twenty four wheat genotypes on soil biochemical and microbial properties. *Plant and Soil* 404 141–155. 10.1007/s11104-016-2833-1

[B21] Corre-Hellou G. Dibet A. Hauggaard-Nielsen H. Crozat Y. Gooding M. Ambus P. (2011). The competitive ability of pea–barley intercrops against weeds and the interactions with crop productivity and soil N availability. *Field Crops Res.* 122 264–272. 10.1016/j.fcr.2011.04.004

[B22] da Rocha U. N. Plugge C. M. George I. van Elsas J. D. van Overbeek L. S. (2013). The rhizosphere selects for particular groups of acidobacteria and verrucomicrobia. *PLoS One* 8:e82443. 10.1371/journal.pone.0082443 24349285 PMC3862674

[B23] de Vries F. Griffiths R. I. Bailey M. Craig H. Girlanda M. Gweon H. S. (2018). Soil bacterial networks are less stable under drought than fungal networks’. *Nat. Commun.* 9:3033. 10.1038/s41467-018-05516-7 30072764 PMC6072794

[B24] Dinno A. (2015). Nonparametric pairwise multiple comparisons in independent groups using Dunn’s test. *Stata J.* 15 292–300. 10.1177/1536867X1501500117

[B25] Edgar R. C. (2010). Search and clustering orders of magnitude faster than BLAST. *Bioinformatics* 26 2460–2461. 10.1093/bioinformatics/btq461 20709691

[B26] Fan K. Weisenhorn P. Gilbert J. A. Chu H. (2018). Wheat rhizosphere harbors a less complex and more stable microbial co-occurrence pattern than bulk soil. *Soil Biol. Biochem.* 125 251–260. 10.1016/j.soilbio.2018.07.022

[B27] Gao L. Liu X.-M. Du Y.-M. Zong H. Shen G.-M. (2019). Effects of tobacco-peanut relay intercropping on soil bacteria community structure. *Ann. Microbiol.* 69 1531–1536. 10.1007/s13213-019-01537-9

[B28] Ghaley B. B. Hauggaard-Nielsen H. Høgh-Jensen H. Jensen E. S. (2005). Intercropping of wheat and pea as influenced by nitrogen fertilization. *Nutr. Cycl. Agroecosys.* 73 201–212. 10.1007/s10705-005-2475-9

[B29] Gómez Expósito R. de Bruijn I. Postma J. Raaijmakers J. M. (2017). Current insights into the role of rhizosphere bacteria in disease suppressive soils. *Front. Microbiol.* 8:2529. 10.3389/fmicb.2017.02529 29326674 PMC5741648

[B30] Gong X. Liu C. Li J. Luo Y. Yang Q. Zhang W. (2019). Responses of rhizosphere soil properties, enzyme activities and microbial diversity to intercropping patterns on the Loess Plateau of China. *Soil Till. Res.* 195:104355. 10.1016/j.still.2019.104355

[B31] Grayston S. J. Wang S. Campbell C. D. Edwards A. C. (1998). Selective influence of plant species on microbial diversity in the rhizosphere. *Soil Biol. Biochem.* 30 369–378. 10.1016/S0038-0717(97)00124-7

[B32] Guiducci M. Tosti G. Falcinelli B. Benincasa P. (2018). Sustainable management of nitrogen nutrition in winter wheat through temporary intercropping with legumes’. *Agron. Sustain. Dev.* 38:31. 10.1007/s13593-018-0509-3

[B33] Guttman D. S. McHardy A. C. Schulze-Lefert P. (2014). Microbial genome-enabled insights into plant–microorganism interactions. *Nat. Rev.Genet.* 15 797–813. 10.1038/nrg3748 25266034

[B34] Hacquard S. Schadt C. W. (2015). Towards a holistic understanding of the beneficial interactions across the populus microbiome. *New Phytol.* 205 1424–1430. 10.1111/nph.13133 25422041

[B35] Hartmann A. Rothballer M. Schmid M. (2008). Lorenz Hiltner, a pioneer in rhizosphere microbial ecology and soil bacteriology research. *Plant Soil* 312 7–14. 10.1007/s11104-007-9514-z

[B36] Herrmann L. Chotte J. L. Thuita M. Lesueur D. (2014). Effects of cropping systems, maize residues, application and N fertilization on promiscuous soybean yields and diversity of native rhizobia in Central Kenya. *Pedobiologia* 57 75–85. 10.1016/j.pedobi.2013.12.004

[B37] Hiltner L. (1903). Beiträge zur Mykorrhizafrage: über die biologische und physiologische Bedeutung der endotrophen Mykorrhiza. *Nat. Wiss. Z. Niederbayern* 1 1–17.

[B38] Hinsinger P. Betencourt E. Bernard L. Brauman A. Plassard C. Shen J. (2011). P for two, sharing a scarce resource: soil phosphorus acquisition in the rhizosphere of intercropped species’. *Plant Physiol.* 156 1078–1086. 10.1104/pp.111.175331 21508183 PMC3135963

[B39] Horner A. Browett S. S. Antwis R. E. (2019). Mixed-cropping between field pea varieties alters root bacterial and fungal communities. *Sci. Rep.* 9:16953. 10.1038/s41598-019-53342-8 31740751 PMC6861290

[B40] Jacquiod S. Puga-Freitas R. Spor A. Mounier A. Monard C. Mougel C. (2020). A core microbiota of the plant-earthworm interaction conserved across soils. *Soil Biol. Biochem.* 144:107754. 10.1016/j.soilbio.2020.107754

[B41] Kalam S. Basu A. Ahmad I. Sayyed R. Z. El-Enshasy H. A. Dailin D. J. (2020). Recent understanding of soil acidobacteria and their ecological significance: a critical review. *Front. Microbiol.* 11:580024. 10.3389/fmicb.2020.580024 33193209 PMC7661733

[B42] Kavamura V. N. Robinson R. J. Hughes D. Clark I. Rossmann M. Soares de Melo I. (2020). Wheat dwarfing influences selection of the rhizosphere microbiome. *Sci. Rep.* 10:1452. 10.1038/s41598-020-58402-y 31996781 PMC6989667

[B43] Kielak A. M. Barreto C. C. Kowalchuk G. A. van Veen J. A. Kuramae E. E. (2016a). The ecology of acidobacteria: moving beyond genes and genomes. *Front. Microbiol.* 7:744. 10.3389/fmicb.2016.00744 27303369 PMC4885859

[B44] Kielak A. M. Cipriano M. A. P. Kuramae E. E. (2016b). Acidobacteria strains from subdivision 1 act as plant growth-promoting bacteria. *Arch. Microbiol.* 198 987–993. 10.1007/s00203-016-1260-2 27339258 PMC5080364

[B45] Knörzer H. Graeff-Hönninger S. Guo B. Wang P. Claupein W. (2009). “The rediscovery of intercropping in China: a traditional cropping system for future chinese agriculture – a review,” in *Climate Change, Intercropping, Pest Control and Beneficial Microorganisms*, ed. Lichtfouse E. (Dordrecht: Springer Netherlands), 13–44. 10.1007/978-90-481-2716-0_3

[B46] Lakshmanan V. Selvaraj G. Bais H. P. (2014). Functional soil microbiome: belowground solutions to an aboveground problem. *Plant Physiol.* 166 689–700. 10.1104/pp.114.245811 25059708 PMC4213098

[B47] Latif S. Bibi S. Kouser R. Fatimah H. Farooq S. Naseer S. (2020). Characterization of bacterial community structure in the rhizosphere of Triticum Aestivum L. *Genomics* 112 4760–4768. 10.1016/j.ygeno.2020.07.031 32712294

[B48] Lemanceau P. Blouin M. Muller D. Moënne-Loccoz Y. (2017). Let the core microbiota be functional. *Trends Plant Sci.* 22 583–595. 10.1016/j.tplants.2017.04.008 28549621

[B49] Lemanceau P. Corberand T. Gardan L. Latour X. Laguerre G. Boeufgras J. (1995). Effect of two plant species, flax (Linum usitatissinum L.) and tomato (Lycopersicon esculentum Mill.), on the diversity of soilborne populations of fluorescent pseudomonads. *Appl. Environ. Microbiol.* 61 1004–1012. 10.1128/AEM.61.3.1004-1012.1995 16534950 PMC1388382

[B50] Lemanceau P. Maron P.-A. Mazurier S. Mougel C. Pivato B. Plassart P. (2015). Understanding and managing soil biodiversity: a major challenge in agroecology. *Agron. Sustain. Dev.* 35 67–81. 10.1007/s13593-014-0247-0

[B51] Li L. Li S.-M. Sun J.-H. Zhou L.-L. Bao X.-G. Zhang H.-G. (2007). Diversity enhances agricultural productivity via rhizosphere phosphorus facilitation on phosphorus-deficient soils’. *Proc. Natl. Acad. Sci. U. S. A.* 104 11192–11196. 10.1073/pnas.0704591104 17592130 PMC1899187

[B52] Li L. Tang C. Rengel Z. Zhang F. (2003). Chickpea facilitates phosphorus uptake by intercropped wheat from an organic phosphorus source. *Plant Soil* 248 297–303.

[B53] Li N. Gao D. Zhou X. Chen S. Li C. Wu F. (2020). Intecropping with potato-onion enhanced the soil microbial diversity of tomato. *Microorganisms* 8:834. 10.3390/microorganisms8060834 32498315 PMC7357159

[B54] Li Q. Chen J. Wu L. Luo X. Li N. Arafat Y. (2018). Belowground interactions impact the soil bacterial community, soil fertility, and crop yield in maize/peanut intercropping systems. *Int. J. Mol. Sci.* 19:622. 10.3390/ijms19020622 29470429 PMC5855844

[B55] Li W. Wu F. (2018). Diversity and co-occurrence patterns of soil bacterial and fungal communities in seven intecropping systems. *Front. Microbiol.* 9:1521. 10.3389/fmicb.2018.01521 30034385 PMC6043683

[B56] Liu H. Roeder K. Wassrman L. (2010). Stability approach to regularization selection (StARS) for high dimensional graphical models. *Adv. Neural. Inf. Process Syst.* 24, 1432–1440. 25152607 PMC4138724

[B57] Liu Y. Yang H. Liu Q. Zhao X. Xie S. Wang Z. (2021). Effect of two different sugarcane cultivars on rhizosphere bacterial communities of sugarcane and soybean upon intercropping. *Front. Microbiol.* 11:596472. 10.3389/fmicb.2020.596472 33519733 PMC7841398

[B58] Ma B. Wang Y. Ye S. Liu S. Stirling E. Gilbert J. A. (2020). Earth microbial co-occurrence network reveals interconnection pattern across microbiomes. *Microbiome* 8:82. 10.1186/s40168-020-00857-2 32498714 PMC7273686

[B59] Maitra S. Hossain A. Brestic M. Skalicky M. Ondrisik P. Gitari H. (2021). Intecropping – a low input agricultural strategy for food and environmental security. *Agronomy* 11:343. 10.3390/agronomy11020343

[B60] Malézieux E. Crozat Y. Dupraz C. Laurans M. Makowski D. Ozier-Lafontaine H. (2009). Mixing plant species in cropping systems: concepts, tools and models. A review. *Agron. Sustain. Dev.* 29 43–62. 10.1051/agro:2007057

[B61] Mamine F. Farès M. (2020). Barriers and levers to developing wheat-pea intercropping in Europe: a review. *Sustainability* 12:6962. 10.3390/su12176962

[B62] McMurdie P. J. Holmes S. (2013). Phyloseq: an R package for reproducible interactive analysis and graphics of microbiome census data. *PLoS One* 8:e61217. 10.1371/journal.pone.0061217 23630581 PMC3632530

[B63] McMurdie P. J. Holmes S. (2014). Waste not, want not: why rarefying microbiome data is inadmissible. *PLoS Comput. Biol.* 10:e1003531. 10.1371/journal.pcbi.1003531 24699258 PMC3974642

[B64] Morriën E. Hannula S. E. L. Snoek B. Helmsing N. R. Zweers H. de Hollander M. (2017). Soil networks become more connected and take up more carbon as nature restoration progresses. *Nat. Commun.* 8:14349. 10.1038/ncomms14349 28176768 PMC5309817

[B65] Murfet I. C. (1973). Flowering in Pisum. Hr, a gene for high response to photoperiod. *Heredity* 31 157–164. 10.1038/hdy.1973.72

[B66] Newman M. E. J. (2003). The structure and function of complex networks. *SIAM Rev.* 45 167–256. 10.1137/S003614450342480

[B67] Offre P. Pivato B. Siblot S. Gamalero E. Corberand T. Lemanceau P. (2007). Identification of bacterial groups preferentially associated with mycorrhizal roots of Medicago truncatula’. *Appl. Environ. Microbiol.* 73 913–921. 10.1128/AEM.02042-06 17142371 PMC1800773

[B68] Oksanen J. Blanchet G. Friendly M. Kindt R. Legendre P. McGlinn D. (2019). *vegan**: Community Ecology Package. R package version 2.5-6.* Available online at: https://CRAN.R-project.org/package=vegan

[B69] Panke-Buisse K. Poole A. C. Goodrich J. K. Ley R. E. Kao-Kniffin J. (2015). Selection on soil microbiomes reveals reproducible impacts on plant function. *ISME J.* 9 980–989. 25350154 10.1038/ismej.2014.196 PMC4817706

[B70] Peiffer J. A. Spor A. Koren V. Jin Z. Tringe S. G. Dangl J. L. (2013). Diversity and heritability of the maize rhizosphere microbiome under field conditions. *Proc. Natl. Acad. Sci. U.S.A* 110 6548–6553. 10.1073/pnas.1302837110 23576752 PMC3631645

[B71] Pelzer E. Bazot M. Makowski D. Corre-Hellou G. Naudin C. Al Rifaï M. (2012). Pea–wheat intercrops in low-input conditions combine high economic performances and low environmental impacts. *Eur. J. Agron.* 40 39–53. 10.1016/j.eja.2012.01.010

[B72] Petric I. Philippot L. Abbate C. Bispo A. Chesnot T. Hallin S. (2011). Inter-laboratory evaluation of the ISO standard 11063 “soil quality — method to directly extract DNA from soil samples”. *J. Microbiol. Methods* 84 454–460. 10.1016/j.mimet.2011.01.016 21256879

[B73] Philippot L. Raaijmakers J. M. Lemanceau P. van der Putten W. H. (2013). Going back to the roots: the microbial ecology of the rhizosphere. *Nat. Rev. Microbiol.* 11 789–799. 10.1038/nrmicro3109 24056930

[B74] Pivato B. Bru D. Busset H. Deau F. Matejicek A. Philippot L. (2017). Positive effects of plant association on rhizosphere microbial communities depend on plant species involved and soil nitrogen level. *Soil Biol. Biochem.* 114, 1–4. 10.1016/j.soilbio.2017.06.018

[B75] Pivato B. Mazurier S. Lemanceau P. Siblot S. Berta G. Mougel C. (2007). Medicago species affect the community composition of arbuscular mycorrhizal fungi associated with roots’. *New Phytol.* 176 197–210. 10.1111/j.1469-8137.2007.02151.x 17803650

[B76] Price M. N. Dehal P. S. Arkin A. P. (2010). FastTree 2 – approximately maximum-likelihood trees for large alignments. *PLoS One* 5:e9490. 10.1371/journal.pone.0009490 20224823 PMC2835736

[B77] Quast C. Pruesse E. Yilmaz P. Gerken J. Schweer T. Yarza P. (2012). The SILVA ribosomal RNA gene database project: improved data processing and web-based tools. *Nucleic Acids Res.* 41 D590–D596. 10.1093/nar/gks1219 23193283 PMC3531112

[B78] R Development Core Team (2014). *R: A Language and Environment for Statistical Computing. R Foundation for Statistical Computing.* Vienna: R Foundation for Statistical Computing.

[B79] Rognes T. Flouri T. Nichols B. Quince C. Mahé F. (2016). VSEARCH: a versatile open source tool for metagenomics. *PeerJ* 4:e2584. 10.7717/peerj.2584 27781170 PMC5075697

[B80] Simonin M. Dasilva C. Terzi V. Ngonkeu E. L. M. Diouf D. Kane A. (2020). Influence of plant genotype and soil on the wheat rhizosphere microbiome: evidences for a core microbiome across eight african and european soils. *FEMS Microbiol. Ecol.* 96:fiaa067. 10.1093/femsec/fiaa067 32275297

[B81] Solanki M. K. Wang Z. Wang F.-Y. Li C.-N. Gupta C. L. Singh R. K. (2020). Assessment of diazotrophic *proteobacteria* in sugarcane rhizosphere when intecropped with legumes (peanut and soybean) in the field. *Front. Microbiol.* 11:1814. 10.3389/fmicb.2020.01814 32849421 PMC7412970

[B82] Song Y. N. Marschner P. Li L. Bao X. G. Sun J. H. Zhang F. S. (2007). Community composition of ammonia-oxidizing bacteria in the rhizosphere of intecropped wheat (Triticum aestivum L.), maize (Zea mays L.), and faba bean (Vicia faba L.). *Biol. Fertil. Soils* 44 307–314. 10.1007/s00374-007-0205-y

[B83] Spor A. Roucou A. Mounier A. Bru D. Breuil M.-C. Fort F. (2020). Domestication-driven changes in plant traits associated with changes in the assembly of the rhizosphere microbiota in tetraploid wheat. *Sci. Rep.* 10:12234. 10.1038/s41598-020-69175-9 32699344 PMC7376052

[B84] Swenson W. Sloan Wilson D. Elias R. (2000). Artificial ecosystem selection. *Proc. Natl. Acad. Sci. U. S. A.* 97 9110–9114. 10890915 10.1073/pnas.150237597 PMC16830

[B85] Takahashi S. Tomita J. Nishioka K. Hisada T. Nishijima M. (2014). Development of a prokaryotic universal primer for simultaneous analysis of bacteria and archaea using next-generation sequencing. *PLoS One* 9:e105592. 10.1371/journal.pone.0105592 25144201 PMC4140814

[B86] Tang X. Placella S. A. Daydé F. Bernard L. Robin A. Journet E. P. (2016). Phosphorus availability and microbial community in the rhizosphere of intercropped cereal and legume along a P-fertilizer gradient. *Plant Soil* 407 119–134. 10.1007/s11104-016-2949-3

[B87] Tang X. Zhang Y. Jiang J. Meng X. Huang Z. Wu H. (2021). Sugarcane/peanut intecropping system improves physicochemical properties by changing N and P cycling and organic matter turnover in root zone soil. *PeerJ* 9:e10880. 10.7717/peerj.10880 33628642 PMC7894120

[B88] Taschen E. Amenc L. Tournier E. Deleporte P. Malagoli P. Fustec J. (2017). Cereal-legume intercropping modifies the dynamics of the active rhizospheric bacterial community. *Rhizosphere* 3 191–195. 10.1016/j.rhisph.2017.04.011

[B89] Taye Z. M. Helgason B. L. Bell J. K. Norris C. H. Vail S. Robinson S. J. (2020). Core and differentially abundant bacterial taxa in the rhizosphere of field grown Brassica napus genotypes: implications for canola breeding. *Front. Microbiol.* 10:3007. 10.3389/fmicb.2019.03007 32010086 PMC6974584

[B90] Theis K. R. Dheilly N. M. Klassen J. L. Brucker R. M. Baines J. F. Bosch T. C. G. (2016). Getting the hologenome concept right: an eco-evolutionary framework for hosts and their microbiomes. *MSystems* 1 e28–e16. 10.1128/mSystems.00028-16 27822520 PMC5069740

[B91] Tkacz A. Bestion E. Bo Z. Hortala M. Poole P. S. (2020). Influence of plant fraction, soil, and plant species on microbiota: a multikingdom comparison. *MBio* 11 e2785–e2719. 10.1128/mBio.02785-19 32019791 PMC7002342

[B92] Turner T. R. Ramakrishnan K. Walshaw J. Heavens D. Alston M. Swarbreck D. (2013). Comparative metatranscriptomics reveals kingdom level changes in the rhizosphere microbiome of plants. *ISME J.* 7 2248–2258. 10.1038/ismej.2013.119 23864127 PMC3834852

[B93] van der Heijden M. G. A. Hartmann M. (2016). Networking in the plant microbiome. *PLoS Biol.* 14:e1002378. 10.1371/journal.pbio.1002378 26871440 PMC4752285

[B94] Vandenkoornhuyse P. Quaiser A. Duhamel M. Le Van A. Dufresne A. (2015). The importance of the microbiome of the plant holobiont. *New Phytol.* 206 1196–1206. 10.1111/nph.13312 25655016

[B95] Wang L. Zou R. Li Y. C. Tong Z. You M. Huo W. (2020). Effect of wheat-Solanum nigrum L. Intecropping on Cd accumulation by plants and soil bacterial community under Cd contaminated soil. *Ecotoxil. Environ. Saf.* 206:111383. 10.1016/j.ecoenv.2020.111383 33002822

[B96] Wei Z. Jousset A. (2017). Plant breeding goes microbial. *Trends Plant Sci.* 22 555–558. 10.1016/j.tplants.2017.05.009 28592368

[B97] Wezel A. Casagrande M. Celette F. Vian J.-F. Ferrer A. Peigné J. (2014). Agroecological practices for sustainable agriculture. A review. *Agron. Sustain. Dev.* 34 1–20. 10.1007/s13593-013-0180-7

[B98] Wickham H. François R. Henry L. Müller K. (2021). *dplyr: A Grammar of Data Manipulation. R Package Version 1.0.6.* Available online at: https://CRAN.R-project.org/package=dplyr

[B99] Wielbo J. (2012). Rhizobial communities in symbiosis with legumes: genetic diversity, competition and interactions with host plants. *Open Life Sci.* 7 363–372. 10.2478/s11535-012-0032-5

[B100] Willey R. W. (1979). Intercropping-its importance and research needs. Part 1. Competition and yield advantage. Part 2. Agronomy and research approaches. *Field Crop Abstracts* 32 73–85.

[B101] Xue Y. Xia H. Christie P. Zhang Z. Li L. Tang C. (2016). Crop acquisition of phosphorus, iron and zinc from soil in cereal/legume intercropping systems: a critical review. *Ann. Bot.* 117 363–377. 10.1093/aob/mcv182 26749590 PMC4765540

[B102] Zhang H. Zeng F. Zou Z. Zhang Z. Li Y. (2017). Nitrogen uptake and transfer in a soybean/maize intercropping system in the Karst region of Southwest China. *Ecol. Evol.* 7 8419–8426. 10.1002/ece3.3295 29075459 PMC5648690

[B103] Zhong Y. Yang Y. Liu P. Xu R. Rensing C. Fu X. (2019). Genotype and Rhizobium inoculation modulate the assembly of soybean rhizobacterial communities. *Plant Cell Environ.* 42 2028–2044. 10.1111/pce.13519 30646427

[B104] Zuo Y. Zhang F. (2009). Iron and zinc biofortification strategies in dicot plants by intercropping with gramineous species. A review. *Agron. Sustain. Dev.* 29 63–71. 10.1051/agro:2008055

