# Supplementary figures and images for: Rhizosphere Bacterial Networks, but Not Diversity, Are Impacted by Pea-Wheat Intercropping
Source: Front Microbiol. 2021 May 28;12:674556. doi: 10.3389/fmicb.2021.674556 (PMC8195745; doi:10.3389/fmicb.2021.674556)

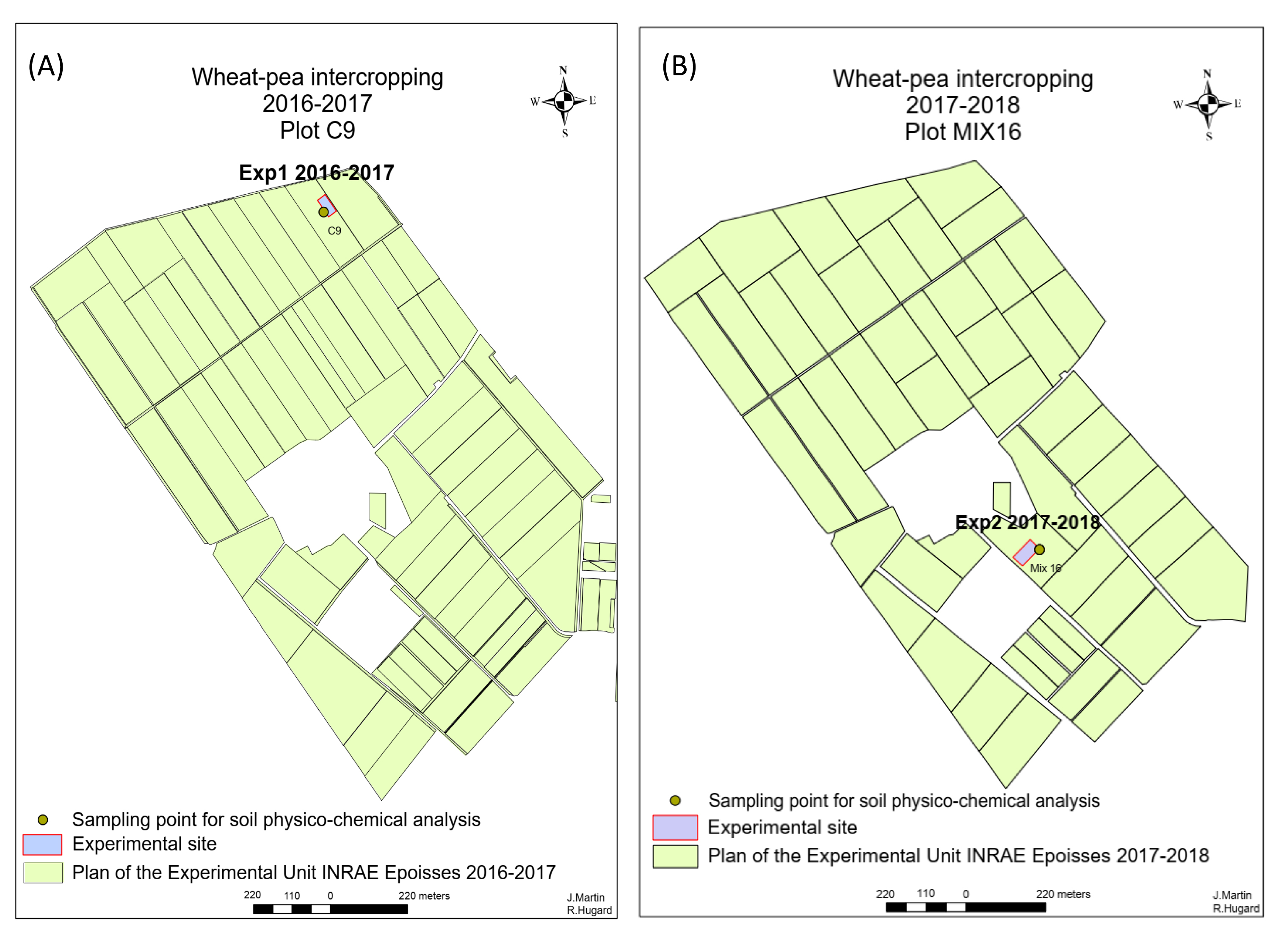

Supplement: Supplementary Figure 1 — Plan of the Experimental Unit INRAE-Epoisses, France (47°14’11.2″ N 5°05’56.1″ E) in panels (A) 2016-2017 and (B) 2017-2018. Blue rectangles with red outline indicate the experimental site in panels (A) 2016-2017 and (B) 2017-2018. The dark green circles with black outline indicate the sampling site for soil physical-chemical analyses. [file Image_1.jpg]

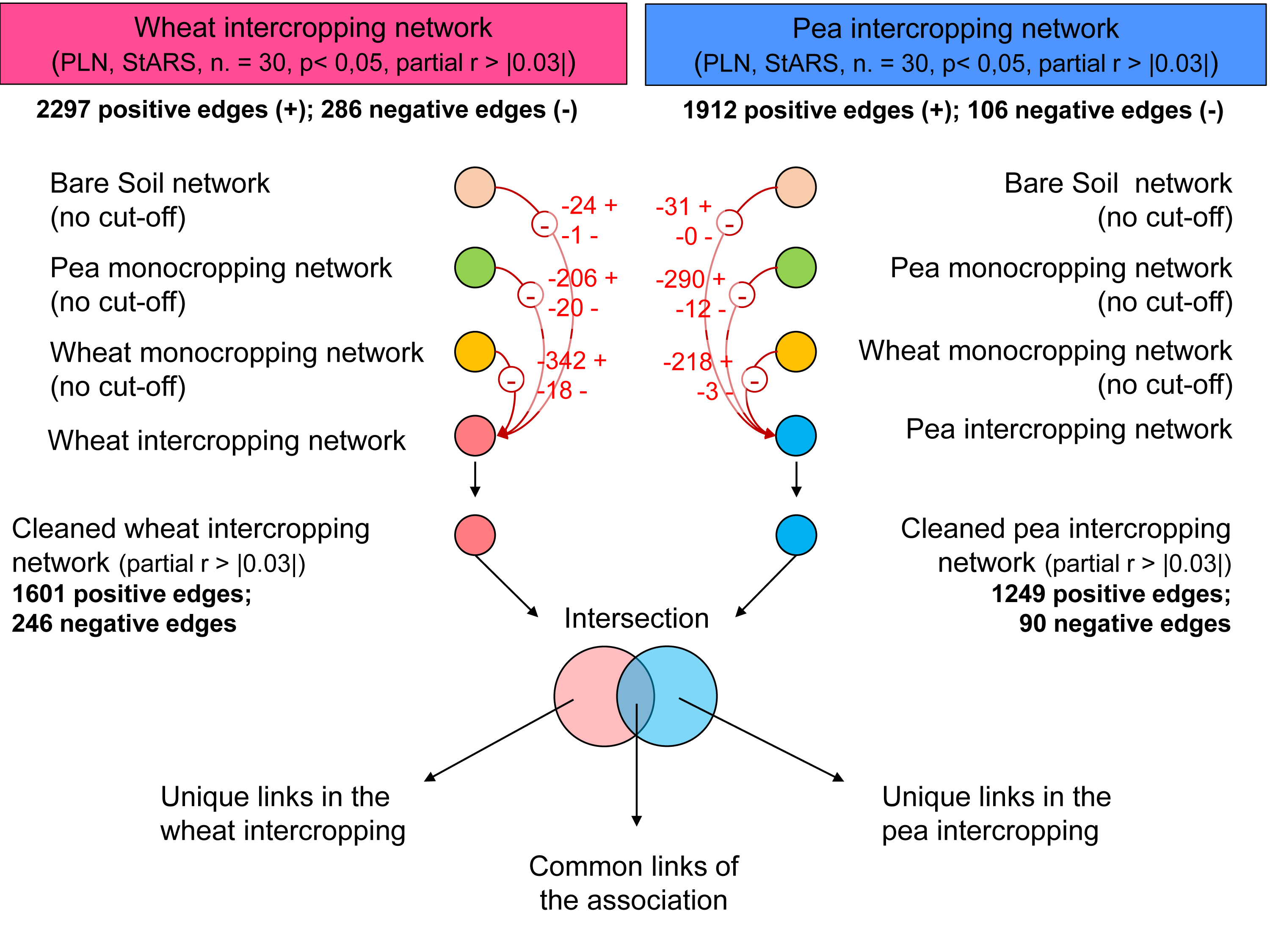

Supplement: Supplementary Figure 2 — Tailored approach to identify OTU correlations that are specific of the intercropping. [file Image_2.tif]

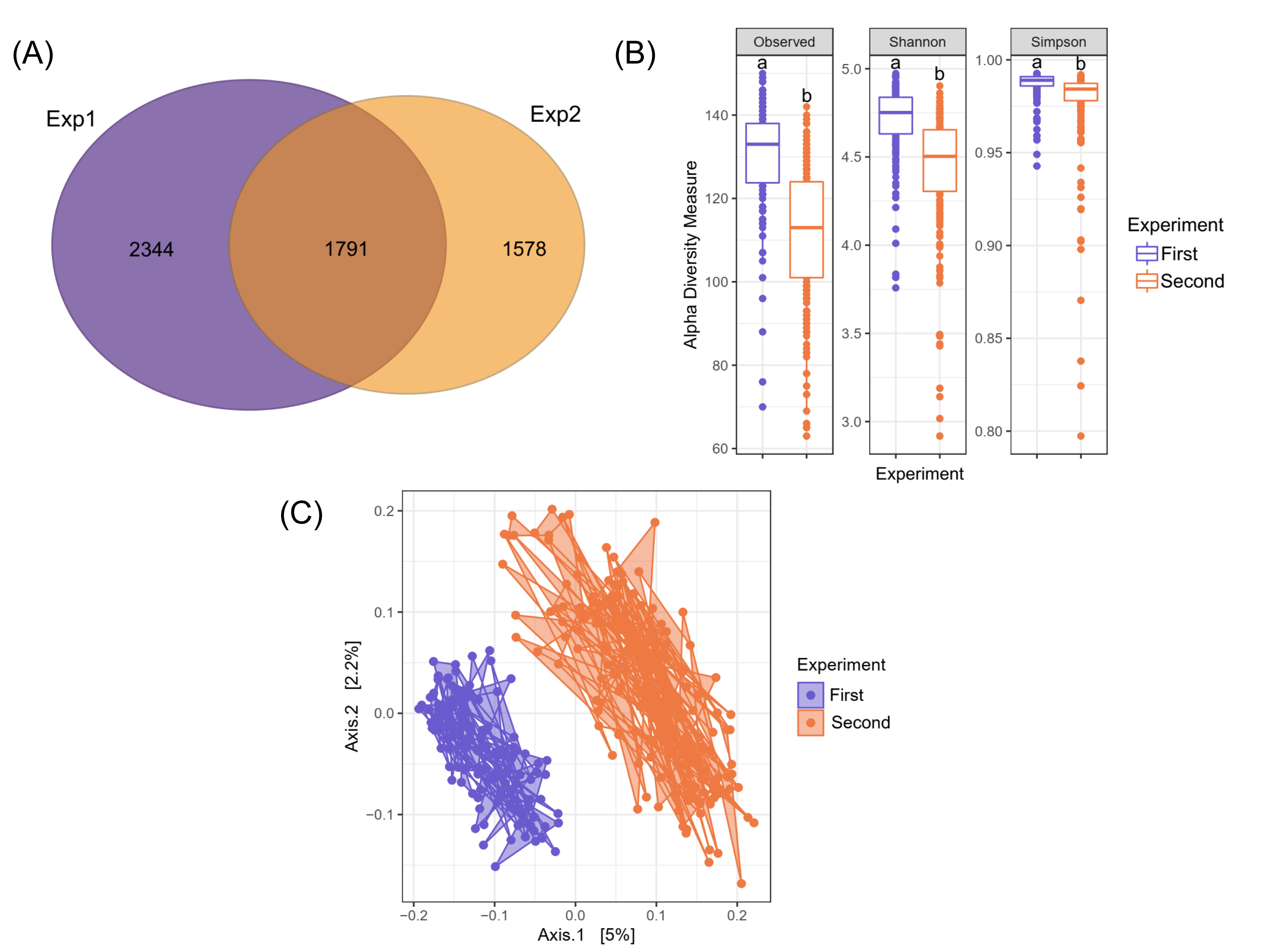

Supplement: Supplementary Figure 3 — Impact of wheat and pea on rhizosphere bacterial community. (A) Box-plots illustrate α-diversity indices (Observed, Shannon, and Simpson) in bacterial communities of wheat (red) and pea (light blue) rhizosphere in the first and the second experiments. Median values and interquartile ranges are indicated in the plots. Different letters indicate significant differences according to Wilcoxon-Mann-Whitney test. (B) Box-plots illustrate α-diversity indices (Observed, Shannon and Simpson) in bacteriobomes of wheat (red) and pea (light blue) rhizosphere corresponding to the pooled dataset of information from the first and second experiments. Median values and interquartile ranges are indicated in the plots. Different letters indicate significant differences according to Wilcoxon-Mann-Whitney test. (C) PCoA (with unweighted UniFrac) showing the β-diversity results of rhizosphere bacterial community profiles of wheat and pea rhizosphere ine the first (First) and the second (Second) experiment. [file Image_3.tif]

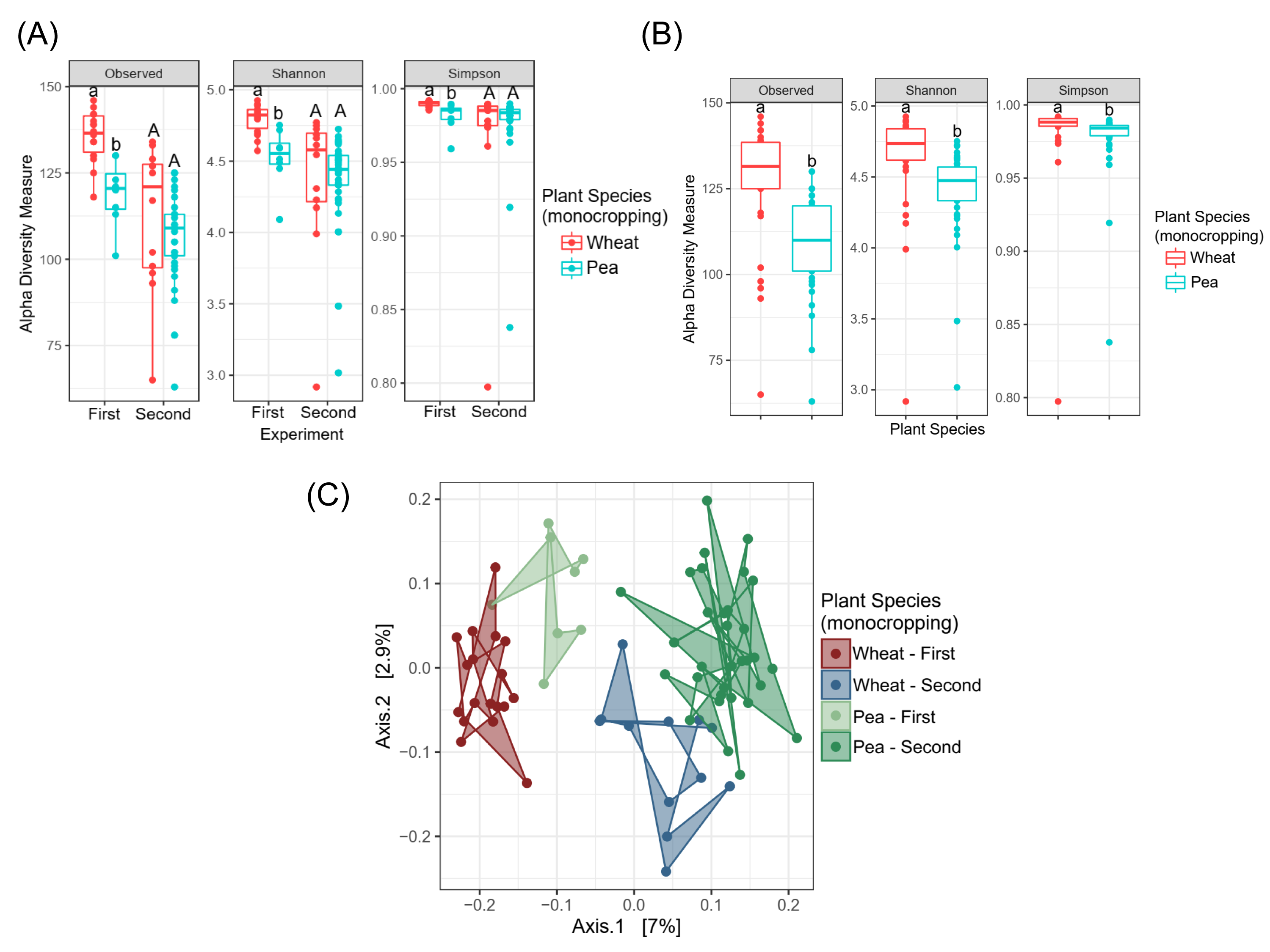

Supplement: Supplementary Figure 4 — Impact of the wheat cultivars in sole cropping on rhizosphere bacterial community. (A) Box-plots illustrating α-diversity indices (Observed, Shannon, and Simpson) in bacterial community of wheat cultivars in the first experiment. Median values and interquartile ranges are indicated in the plots. (B) PCoA (with unweighted UniFrac) showing the β-diversity results of rhizosphere microbiota profiles of wheat cultivars corresponding to the first experiment. [file Image_4.tif]

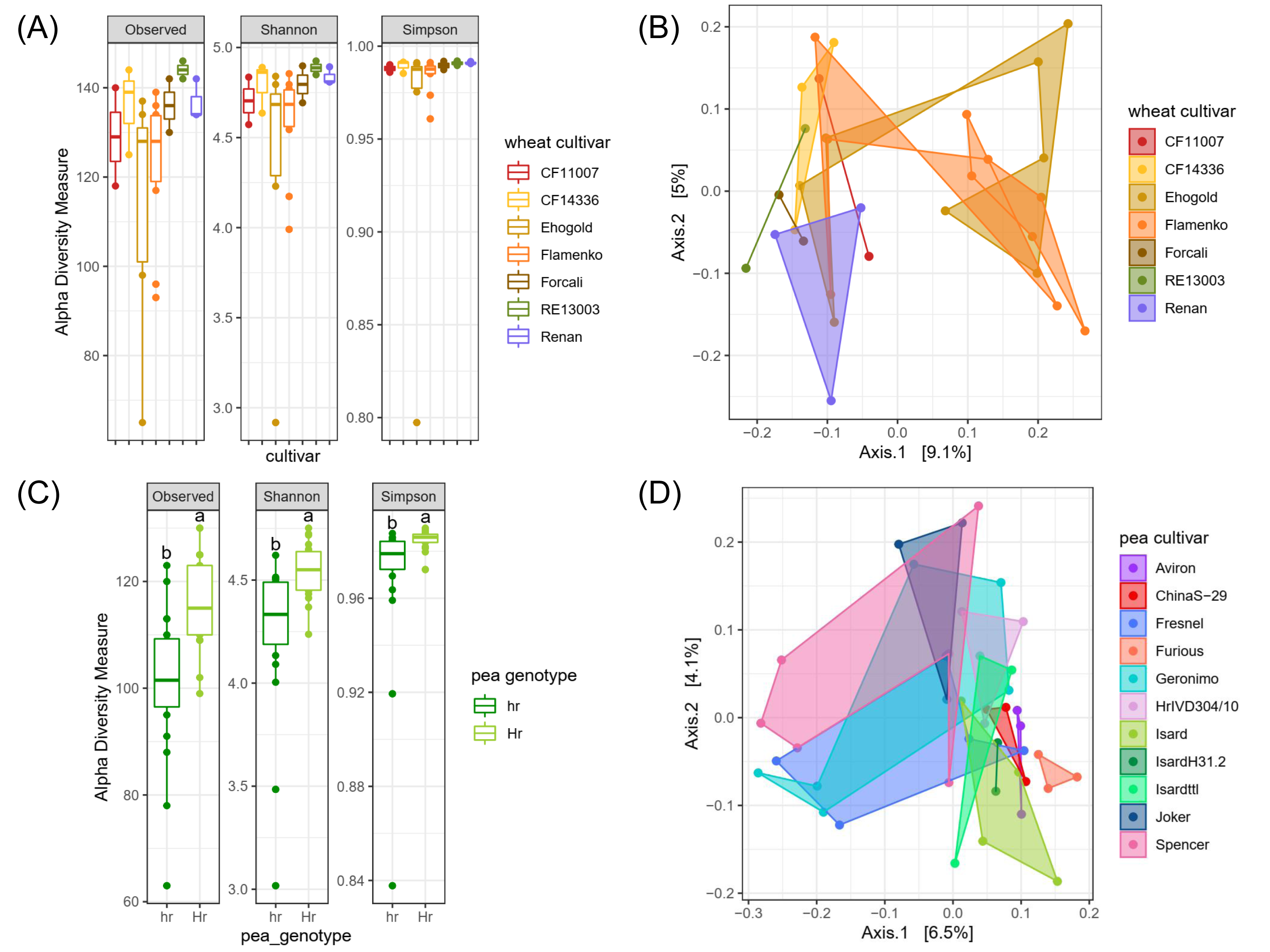

Supplement: Supplementary Figure 5 — Impact of the pea cultivars in sole cropping on rhizosphere bacterial community. (A) Box-plots illustrate α-diversity indices (Observed, Shannon and Simpson) in bacterial community of pea cultivars in the second experiment. Median values and interquartile ranges are indicated in the plots. (B) Box-plots illustrating α-diversity indices (Observed, Shannon and Simpson) in bacterial community of pea hr and Hr genotypes corresponding to the pooled dataset. Median values and interquartile ranges are indicated in the plots. Different letters indicate significant differences according to Wilcoxon-Mann-Whitney test. (C) PCoA (with unweighted UniFrac) showing the β-diversity results of rhizosphere microbiota profiles of pea cultivars corresponding to the first experiment. [file Image_5.tif]

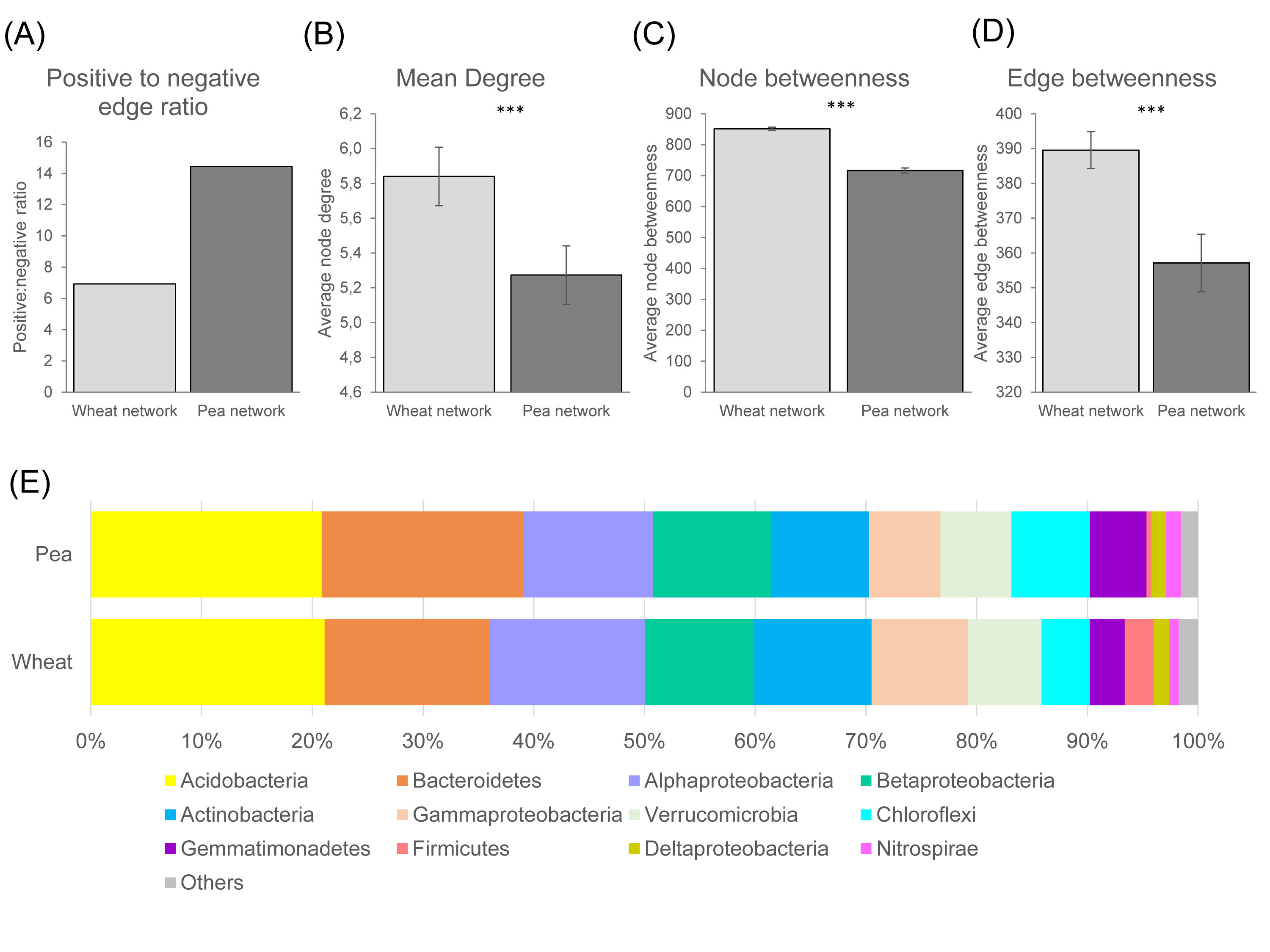

Supplement: Supplementary Figure 6 — Comparison of the architecture characteristics of co-occurring network between intercropped wheat and pea networks: (A) positive to negative edge ratio, (B) mean degree, (C) node betweenness, and (D) edge betweenness. Significant differences were indicated by ∗∗∗, according to Wilcoxon-Mann-Whitney test (p < 0.05). (E) OTUs affiliations in intercropped wheat and pea networks. [file Image_6.tif]
